# An NO Donor Approach to Neuroprotective and Procognitive Estrogen Therapy Overcomes Loss of NO Synthase Function and Potentially Thrombotic Risk

**DOI:** 10.1371/journal.pone.0070740

**Published:** 2013-08-16

**Authors:** Lawren VandeVrede, Ramy Abdelhamid, Zhihui Qin, Jaewoo Choi, Sujeewa Piyankarage, Jia Luo, John Larson, Brian M. Bennett, Gregory R. J. Thatcher

**Affiliations:** 1 Department of Medicinal Chemistry and Pharmacognosy, University of Illinois College of Pharmacy, University of Illinois at Chicago, Chicago, Illinois, United States of America; 2 Department of Psychiatry, Neuropsychiatric Institute, University of Illinois at Chicago, Chicago, Illinois, United States of America; 3 Department of Biomedical and Molecular Sciences, Faculty of Health Sciences, Queen's University, Kingston, Ontario, Canada; University of Padova, Italy

## Abstract

Selective estrogen receptor modulators (SERMs) are effective therapeutics that preserve favorable actions of estrogens on bone and act as antiestrogens in breast tissue, decreasing the risk of vertebral fractures and breast cancer, but their potential in neuroprotective and procognitive therapy is limited by: 1) an increased lifetime risk of thrombotic events; and 2) an attenuated response to estrogens with age, sometimes linked to endothelial nitric oxide synthase (eNOS) dysfunction. Herein, three 3^rd^ generation SERMs with similar high affinity for estrogen receptors (ERα, ERβ) were studied: desmethylarzoxifene (DMA), FDMA, and a novel NO-donating SERM (NO-DMA). Neuroprotection was studied in primary rat neurons exposed to oxygen glucose deprivation; reversal of cholinergic cognitive deficit was studied in mice in a behavioral model of memory; long term potentiation (LTP), underlying cognition, was measured in hippocampal slices from older 3×Tg Alzheimer's transgenic mice; vasodilation was measured in rat aortic strips; and anticoagulant activity was compared. Pharmacologic blockade of GPR30 and NOS; denudation of endothelium; measurement of NO; and genetic knockout of eNOS were used to probe mechanism. Comparison of the three chemical probes indicates key roles for GPR30 and eNOS in mediating therapeutic activity. Procognitive, vasodilator and anticoagulant activities of DMA were found to be eNOS dependent, while neuroprotection and restoration of LTP were both shown to be dependent upon GPR30, a G-protein coupled receptor mediating estrogenic function. Finally, the observation that an NO-SERM shows enhanced vasodilation and anticoagulant activity, while retaining the positive attributes of SERMs even in the presence of NOS dysfunction, indicates a potential therapeutic approach without the increased risk of thrombotic events.

## Introduction

In addition to developmental functions, estrogens have been found to reduce incidence of coronary heart disease [1], maintain bone mineral density, and, in the CNS, promote neuronal survival [2] and hippocampal neurogenesis [3], [4]. Neuro-imaging studies reveal that estrogen therapy improves cerebral blood flow and performance in hippocampal-dependent memory tasks in women age 55 and older [5], [6]. Other observational studies have found that estrogen helps alleviate age-related cognitive decline by preserving executive function in young and postmenopausal women [7]. Meta-analysis suggested that the risk of Alzheimer's disease (AD) could be reduced by estrogen replacement therapy (ERT) by as much as 34% [8], [9]. Although the Women's Heath Initiative (WHI) study attempted to investigate the impact of ERT on dementia, the study concluded prematurely due to reported risk of stroke and breast cancer [10], [11]. These findings have led several authors to the conclusion that estrogen therapy remains a treatment or prophylactic option for cognitive impairment and AD, if carcinogenic and thromboembolic effects can be ameliorated [12], [13].

Raloxifene (Evista) is a second generation SERM used clinically for the treatment of osteoporosis in postmenopausal women, which acts as an antiestrogen in breast and endometrial tissues and has been shown to reduce the lifetime risk of vertebral fractures and breast cancer [14], [15], [16], [17]. Additionally, clinical trials showed a trend towards decreased risk for cognitive impairment [18], [19], with no effect on coronary events, although these effects must be balanced against raloxifene's known increased lifetime risk of thromboembolic events [20]. Raloxifene has also been found to enhance levels of the vasodilator NO through actions on endothelial nitric oxide synthase (eNOS) [21], [22], [23]; however, age-related attenuated eNOS activity has been speculated as a cause of increased thromboembolic events in postmenopausal women [24]. Since NO is known to inhibit thrombus formation through inhibition of platelet recruitment, adhesion and aggregation [25], it appeared worthwhile to test the novel concept that an NO-donor SERM (NO-SERM) could abrogate or circumvent adverse events linked to eNOS dysfunction in postmenopausal women.

The activation of NO signaling in combination with estrogen therapy may be of use in an aging population including AD patients, since eNOS activity may decrease with age [26], [27]. This loss of activity may be associated with the critical period hypothesis, wherein women who are ≥10 years post-menopause are less responsive or nonresponsive to the neuroprotective and procognitive effects of estrogens [28], [29], [30], [31], [32], [33]. It is likely that multiple pathways contribute to the attenuated estrogen response, and based upon mechanistic studies, these may include signaling via estrogen receptors (ER), GPR30, and eNOS [34], [35], [36] (for review see [37]).

The development of the next generation SERM, arzoxifene, was driven by the need to improve on the poor bioavailability of raloxifene [38], [39]. Arzoxifene, is a prodrug of desmethylarzoxifene (DMA) that differs from raloxifene by only one atom, and retains efficacy in reducing the risk of vertebral fracture and invasive breast cancer in postmenopausal women, but failed to demonstrate a significant improvement in cognitive function [40], [41]. To test the NO-SERM concept, DMA was compared to an analog, FDMA, and the NO-donating derivative, NO-DMA (File S1), in several model systems reflecting neuroprotection, cognition, vasodilation, and potential antithrombotic actions. These systems were probed pharmacologically with physical and genetic depletion of eNOS, revealing key roles for GPR30 and NO in the therapeutic actions of SERMs. It was predicted that NO-DMA would provide the benefits of DMA in the presence of eNOS challenge and provide additional therapeutically relevant antithrombotic effects.

Herein we support the hypothesis that SERM neuroprotection is mediated through a GPR30-dependent mechanism, and extend these results to show that GPR30 mediates the actions of SERMs in restoration of synaptic transmission studied in the 3×Tg transgenic AD mouse at an older age than previously reported. We also demonstrate that the procognitive and vasodilator effects of SERMs are dependent on intact eNOS signaling, whereas NO-SERMs preserve action in models where NOS signaling is impaired. This is the first report of an NO-SERM that has potential to act as a vasodilator and antithrombotic agent and is able to reverse deficits in synaptic transmission and memory in mouse models, while preserving ER binding and retaining efficacy in the face of attenuated eNOS activity.

## Results

### Synthesis of an NO-SERM retaining nanomolar binding affinity for ER

Raloxifene and DMA are structurally related SERMs acting as antagonists of estrogen action in mammary and endometrial cells. We have shown that derivatives and analogues of DMA can be synthesized that retain antagonist activity [42], [43], [44], [45], [46]. DMA derivatives and analogues with ER antagonist activity in breast and endometrium would be predicted to reduce the incidence of breast cancer in the clinic, as is observed with raloxifene. It was anticipated based on our previous manipulations of DMA [47], [48], that modification of DMA to introduce NO-donor properties could be accomplished without loss of ER binding and ER antagonist actions. ER binding affinity was measured by competitive E_2_ displacement as previously described [45]. The IC_50_ values for ERα were 7.8±1.9 nM, 17±0.6 nM, and 21.4±6.7 nM for DMA, FDMA and NO-DMA, respectively, while IC_50_ values for ERβ were 9.6±1.9 nM, 16±0.8 nM, and 24.4±9.6 nM, respectively. Therefore, DMA, FDMA, and NO-DMA represent chemical probes with similar and high affinity for both classical estrogen receptors ERα and ERβ. ER-mediated transcriptional activity was measured in the Ishikawa endometrial cancer cell line using the method previously described [46], demonstrating that NO-DMA acts an antiestrogen with potency (IC_50_ = 3.9±1.3 nM) similar to that of DMA (0.1±0.1 nM) and FDMA (1.4±0.4 nM).

### Neuroprotection is mediated through GPR30 activation, independent of ERα and NOS

**Figure 1:**
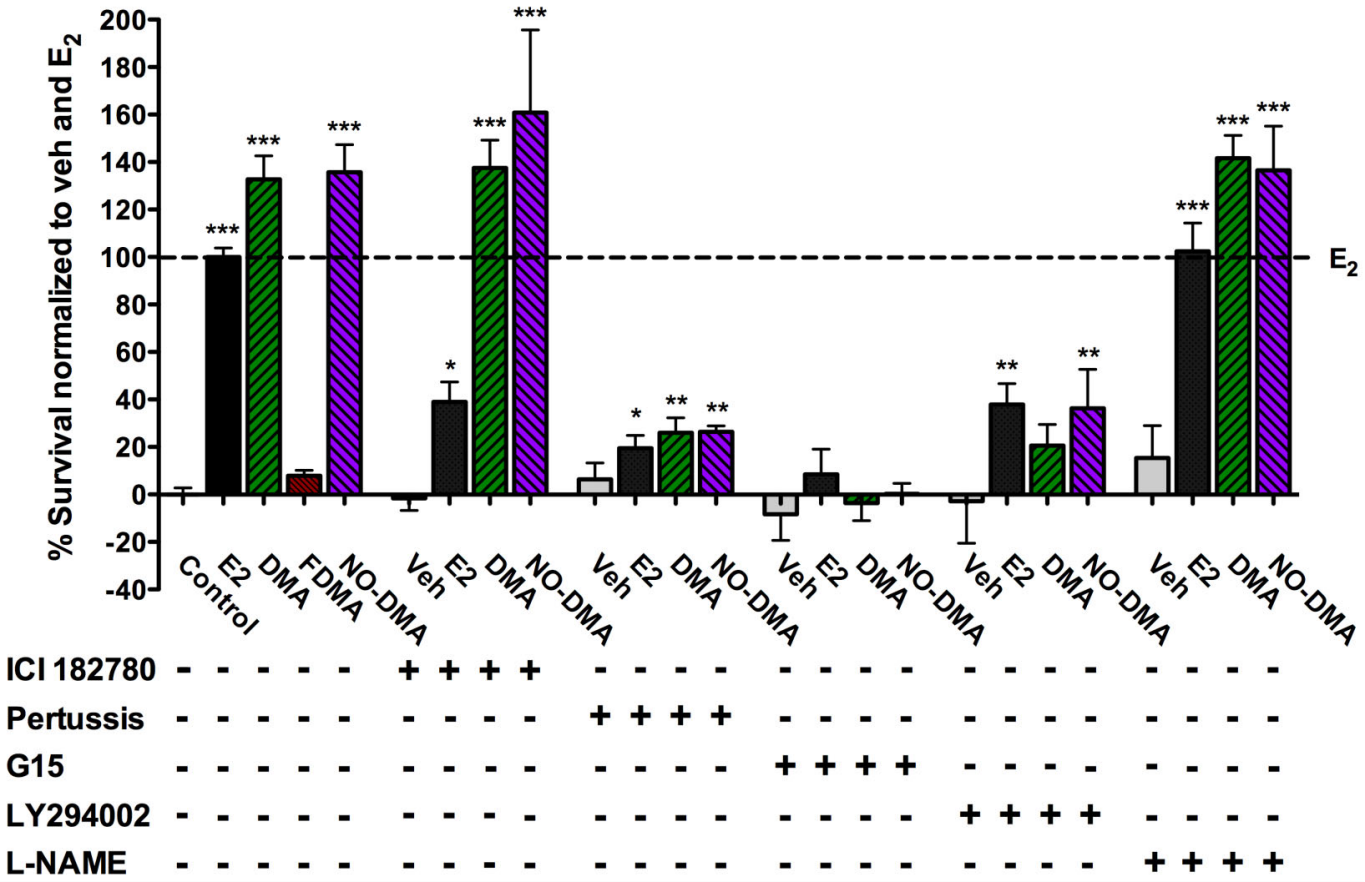
SERM-elicited neuroprotection in primary cortical neurons exposed to OGD is GPR30 dependent and retained by NO-DMA. Primary neuronal cultures were subjected to 2 h OGD with compounds added at the start of OGD and inhibitors added 45 min prior to OGD. Cell survival was measured at 24 h. Use of pathway-selective inhibitors indicates that neuroprotection of DMA and NO-DMA is mediated through PI3K-dependent GPR30 signaling in an ER- and NOS-independent manner. Data show mean and S.E.M. normalized to veh. control and estradiol (n = 6); * = p<0.05, ** = p<0.01, *** = p<0.001 compared to untreated vehicle control using one-way ANOVA with Dunnett's post hoc test within each treatment group; no blocker F_(4,67)_ = 169.5, p<0.0001; ICI 182780 F_(4,61)_ = 58.65, p<0.0001; pertussis F_(4,61)_ = 6.78, p = 0.0001; G15 F_(4,61)_ = 0.63, p = 0.64; LY294002 F_(4,61)_ = 6.29, p<0.001; L-NAME F_(4,61)_ = 89.33, p<0.0001.

It was predicted that NO-DMA would retain the neuroprotective actions of DMA, since previously we have shown that the neuroprotective actions of raloxifene and DMA are GPR30-dependent and mediated via PI3K/Akt signaling in primary neuronal culture in the oxygen glucose deprivation (OGD) assay, a composite model of ischemia-reperfusion injury [47]. A role for NO was not predicted, since in this model, neuroprotection is generally not seen for simple nitrate NO-donors (data not shown). Twenty-four hours after initiation of glucose deprivation, 100 nM NO-DMA was observed to elicit robust neuroprotection identical to DMA, as measured by MTT (Fig. 1) and normalized to estradiol (E2, 10 nM, 100%) and vehicle (0%). Blockade of classical ERα signaling by ICI 182780 (100 nM) did not block this effect. However, both pertussis toxin (100 ng/mL), a G protein coupled receptor blocker, and G15 (100 nM), a selective GPR30 antagonist [49], blocked the neuroprotective activity of NO-DMA and DMA. LY294002 (10 µM), a selective PI3K inhibitor, attenuated neuroprotective activity, whereas L-NAME (100 µM), a non-selective NOS antagonist, did not, supporting the hypothesis that NO-DMA, like DMA, signals through the PI3K/Akt pathway downstream of GPR30. The ineffectiveness of the high affinity ERα ligand, FDMA, in this paradigm is compatible with the inability of this SERM subtype to activate GPR30 signaling, which we have previously reported [47].

In this model, signaling via NOS downstream of PI3/Akt is not indicated. However, NO-DMA retains the GPR30-dependent neuroprotective activity of DMA, independent of NO.

### DMA and NO-DMA restore memory, after cholinergic challenge, via NO release

**Figure 2:**
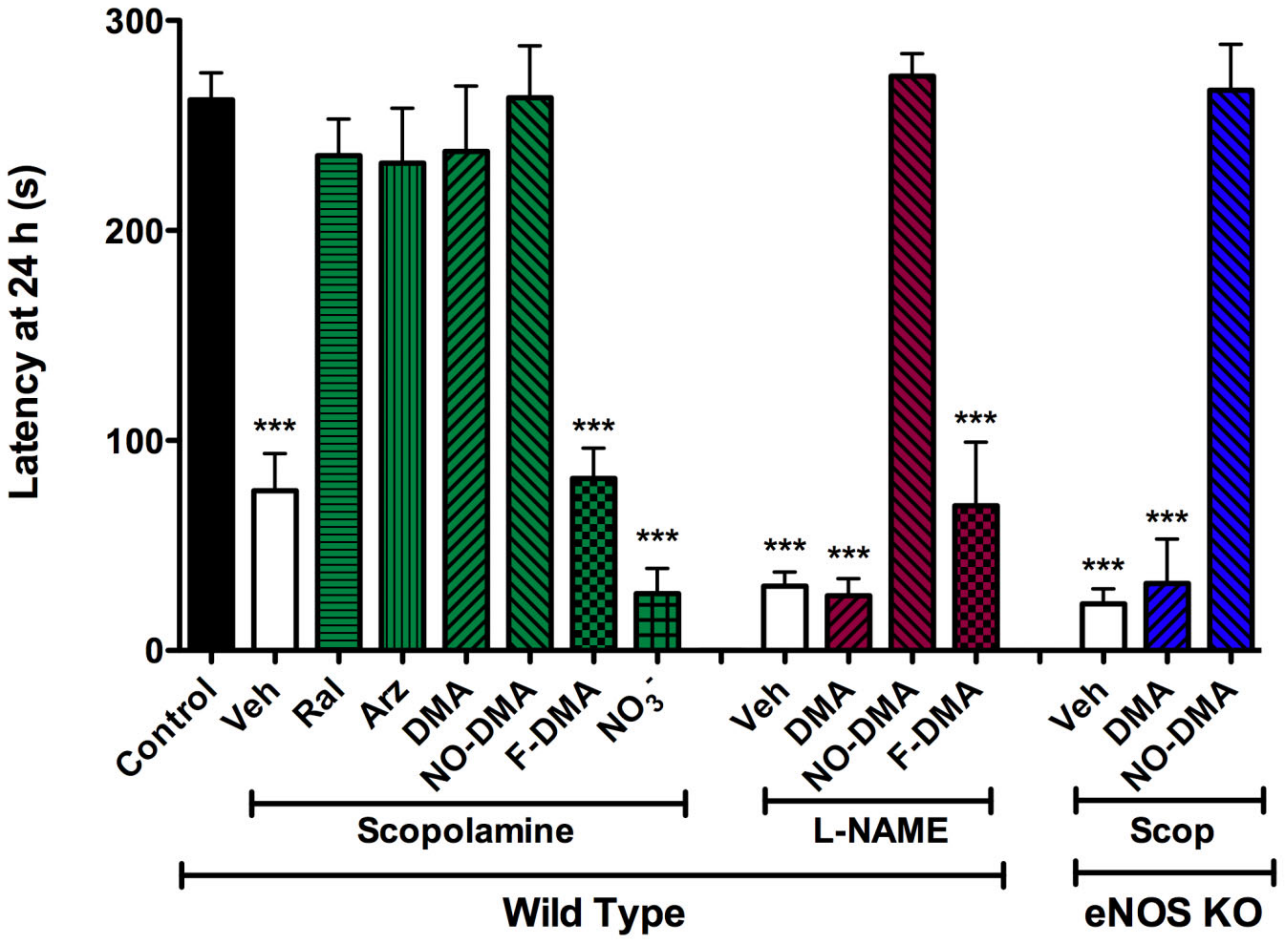
SERM reversal of memory deficits in WT mice is retained by NO-DMA in eNOS (−/−) mice. Amnestic memory deficit was induced by i.p. injection of either scopolamine (1 mg/kg) or L-NAME (50 mg/kg) 30 min prior to training in C57Bl/6 male mice. SERMs (2 mg/kg) were given 20 min prior to training and latency was assessed 24 h after training with animals being removed from the task if latency >300 s. All SERMs, except F-DMA, restored scopolamine-induced deficits in STPA in C57Bl/6 animals. Against L-NAME-induced deficit, only NO-DMA showed efficacy in reversing memory deficits. In eNOS (−/−) animals subject to scopolamine-induced amnesia, only NO-DMA showed efficacy. Data show mean and S.E.M. (n = 4–10); *** = p<0.001 compared to non-insult wild type vehicle control using one-way ANOVA with Dunnett's post hoc test; F_(14,124)_ = 29.26, p<0.0001.

To investigate the procognitive effects of SERMs and NO-DMA in an *in vivo* behavioral model of memory, step-through passive avoidance (STPA) was used in 6–8 month old male C57Bl/6 mice treated with the muscarinic antagonist scopolamine (1 mg/kg) 30 min prior to training. In this assay, memory is tested 24 h after training and since drugs are only administered during training, any side effects, such as sedation, are highly unlikely to confound the testing results. Furthermore, criteria for training are uniform across all treatment groups. Previously, we have shown NO-donors to reverse cognitive deficits induced by attenuation of cholinergic signaling [50], [51], [52], [53]. In animals administered scopolamine to induce amnesia, all treatments except FDMA and inorganic nitrate induced complete reversal of the memory deficit, with NO-DMA and DMA showing equi-efficacious activity (Fig. 2).

To isolate the procognitive effects due to NO production from NOS, versus direct release of NO from NO-DMA, experiments were repeated after administration of the nonselective NOS inhibitor L-NAME in place of scopolamine. L-NAME treatment resulted in a cognitive deficit that was reversed by NO-DMA, but not by DMA. To definitively ascribe the actions of DMA to activation of the endothelial isoform of NOS, experiments were conducted with eNOS(−/−) mice. The scopolamine-induced memory deficit in eNOS KO mice was not influenced by treatment with DMA, whereas NO-DMA again significantly improved memory measured by STPA. These observations strongly implicate eNOS as mediating the procognitive mechanism of action of SERMs and demonstrate the ability of an NO-SERM to circumvent loss of eNOS activity.

### LTP restoration in an Alzheimer's mouse model is GPR30-dependent

**Figure 3:**
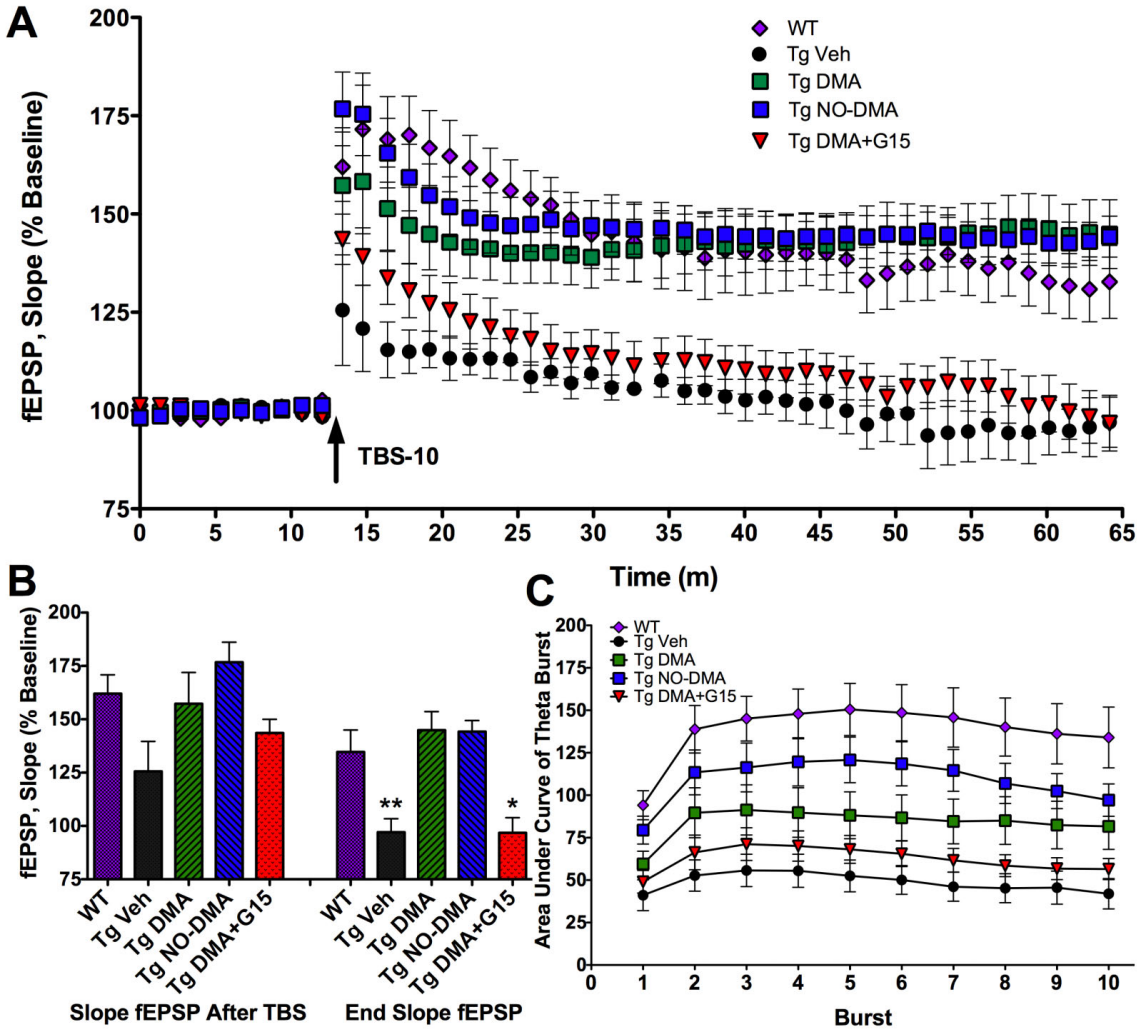
Reversal of LTP deficit in aged 3×Tg mice by SERMs is GPR30 dependent. LTP was measured after TBS in the CA1 region of hippocampal sections from 16 month male 3×Tg mice or WT controls. Test compounds (SERMs 100 nM; G15 100 nM) were added 30 min prior to TBS. (**A, B**) DMA and NO-DMA restored deficits in LTP to WT levels and G15 blocked the actions of DMA. (**C**) Secondary analysis of theta bursts indicate action both during induction and stabilization of LTP, through a GPR30 dependent mechanism. Data show mean and S.E.M. normalized to baseline (n = 4–9); for end fEPSP: * = p<0.05, ** = p<0.01 compared to wild type transgenic background controls using one-way ANOVA with Dunnett's post hoc test; F_(4,32)_ = 8.21, p = 0.0001.

Restoration of LTP via NOS was predicted to be one mechanism underlying the procognitive actions of DMA and NO-DMA, since activation of NO/cGMP signaling has been shown to restore LTP in young APP/PS1 AD transgenic mice [54], [55]. LTP in the CA1 field of the hippocampus is a well-studied cellular model for learning and memory. To measure the effect of DMA and NO-DMA in reversing deficits in synaptic plasticity, LTP was induced with a theta burst stimulation (TBS) protocol at Schaffer/commissural fiber synapses in the CA1 field of hippocampal slices from 16-month old male 3×Tg mice. These mice have been shown to have a marked deficit in LTP that becomes apparent at 6 months of age [56]. At 16 months, an advanced age at which Alzheimer's-like neuropathology is well developed in these animals, studies on LTP have not previously been reported. Nevertheless, we observed a robust, reproducible deficit in LTP in these aged transgenic mice, with field excitatory post-synaptic potentials (fEPSP) showing an end fEPSP of 97.0±6.4% of baseline at 45 min post-TBS, compared to 134.7±10.3% observed in the wild type (WT) transgenic background controls (Fig. 3A,B).

Addition of DMA (100 nM) to the aCSF perfusate 30 min prior to induction of LTP resulted in significant reversal of the LTP deficit to an end fEPSP of 144.9±8.7% (Fig. 3A,B). NO-DMA (100 nM) had effects similar to DMA, with an end fEPSP of 144.2±5.2% of baseline, with a trend towards increased potentiation over untreated transgenics seen immediately after TBS (Fig. 3A,B). Finally, addition of the GPR30 selective antagonist G15 (100 nM) blocked the action of DMA, with end fEPSP approaching levels of untreated transgenics (96.8±7.1%). G15 alone was without effect on hippocampal slices from C57Bl/6 mice in the absence of DMA (Figure S1). These observations suggest that the GPR30 receptor is critical for the LTP-enhancing effects of SERMs. Preliminary data from using a soluble guanylyl cyclase (sGC) inhibitor suggest that the activity of DMA in 3×Tg hippocampus is also sGC and NOS-dependent (Figure S2).

To determine if compounds enhanced LTP by altering physiological responses during LTP induction, such as NMDA receptor-mediated currents or downstream events involved in expression or stabilization, the postsynaptic responses to theta bursts were quantified as previously described [57], [58]. The enhancement of subsequent bursts was augmented by DMA and NO-DMA but not by DMA+G15 (Fig. 3C), suggesting that SERMs may act, at least in part, by enhancing depolarization and NMDA receptor activity during TBS, in addition to downstream actions on the signaling events that lead to stabilization of the LTP response.

In summary, NO-DMA retained the activity of DMA in a model of AD in reversing deficits in synaptic function, and the activity of DMA was GPR30-dependent.

### Vascular relaxation induced by SERMs is retained by NO-SERM in the absence of eNOS

It was predicted that removal of eNOS function, either via inhibition or denuding the endothelium would attenuate vasodilation induced by SERMs. It was also predicted that in the absence of eNOS, the response to an NO-SERM would simply be a right-shift in the dose-response curve. The vasodilator activity of SERMs and NO-SERMs was assessed using isolated aortic ring preparations.

**Figure 4:**
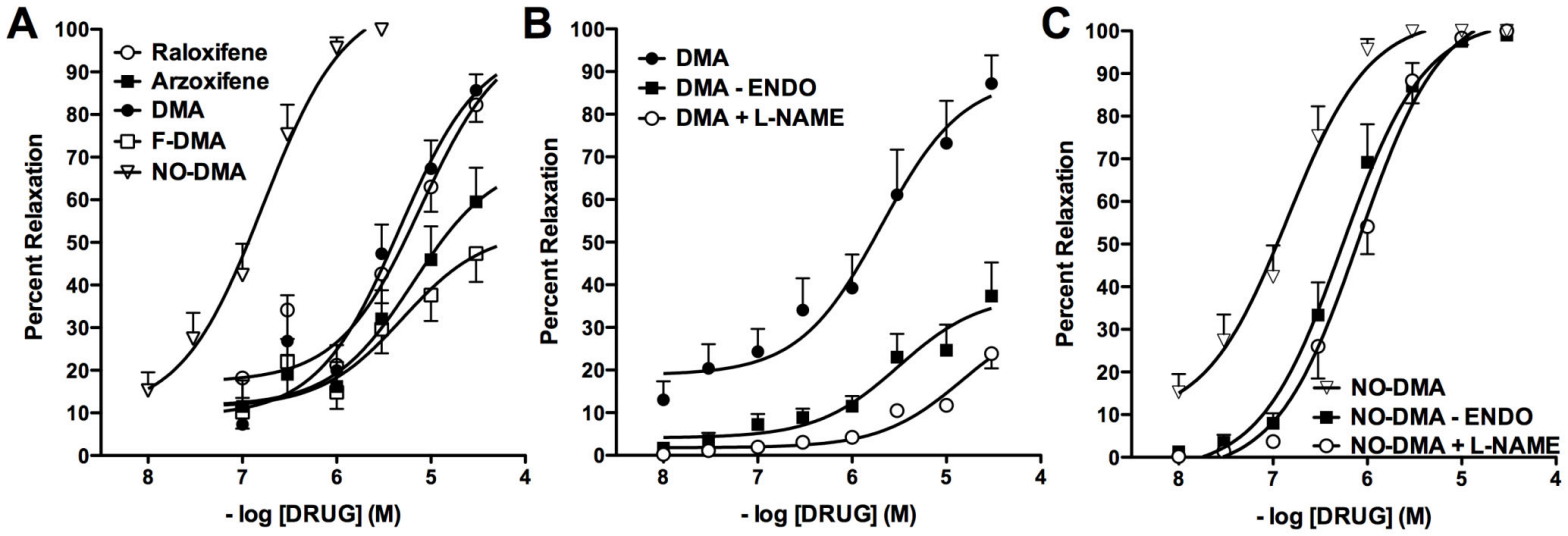
Relaxation of isolated aortic rings by SERMs and NO-SERM. (**A**) The EC_50_ values for relaxation were not significantly different for raloxifene, arzoxifene, DMA, and FDMA (p>0.05, one-way ANOVA and Newman-Keul's post-hoc test), whereas for NO-DMA potency was significantly different from all other SERMs (F_(4,43)_ = 4.085, p<0.01). The maximal relaxation responses for arzoxifene and FDMA were significantly less than those for DMA and raloxifene (F_(3, 37)_ = 11.77 p<0.05, one-way ANOVA and Newman-Keul's post-hoc test). Each value represents the mean ± S.E.M. (n = 7–13). (**B**) Removal of the endothelium or inhibition of NOS with L-NAME reduced the maximal relaxation response to DMA (F_(2, 18)_ = 28.22, p<0.001, one-way ANOVA and Newman-Keuls post-hoc test). Each value represents the mean ± S.E.M. (n = 7). (**C**) The EC_50_ values for relaxation were significantly increased in the presence of L-NAME or after endothelium removal (F_(2,18)_ = 7.753, p<0.05, one-way ANOVA and Newman-Keuls post-hoc test). Each value represents the mean ± S.E.M. (n = 7).

All SERMs tested exhibited dose-dependent relaxation, with raloxifene, DMA and NO-DMA showing efficacy approaching 100% relaxation (Fig. 4A). The following EC_50_ values (mean, S.D.) were calculated but not found to be significantly different: raloxifene, 4.0±2.9 µM; arzoxifene, 3.3±2.5 µM; DMA, 3.5±2.5 µM; FDMA, 2.4±1.2 µM; suggesting equal potency as vasodilators among these SERMs. NO-DMA was designed to provide two mechanisms of vasodilation, via NOS activation and direct release of NO. Compatible with this expectation, NO-DMA was more potent than DMA, inducing maximal relaxation with at least ten-fold increased potency (EC_50_ = 130±90 nM).

Physical removal of the endothelium or inhibition of NOS by L-NAME resulted in a significant decrease in the efficacy of DMA, indicating a significant role for eNOS in mediating the vasodilator activity of DMA (Fig. 4B). Thus, SERMs induce relaxation in the aortic ring model, largely dependent on intact eNOS signaling and an intact endothelium. In endothelium-denuded tissues and in tissues treated with L-NAME, NO-DMA potency was also reduced: EC_50_ values of 0.90±0.69 µM, and 1.0±0.54 µM, respectively (Fig. 4C). The right shift in concentration-response reflects the loss of eNOS mediated, SERM-dependent vasodilation; however, NO-DMA potency, with the ablation of eNOS activity, was equivalent to DMA potency with intact eNOS activity. Therefore, NO-DMA was shown to have both endothelium-dependent and -independent vasodilator actions, the latter mediated through NO bioactivity inherent to an NO-SERM.

### NO generation by SERMs is retained by NO-SERM in the absence of eNOS function

**Figure 5:**
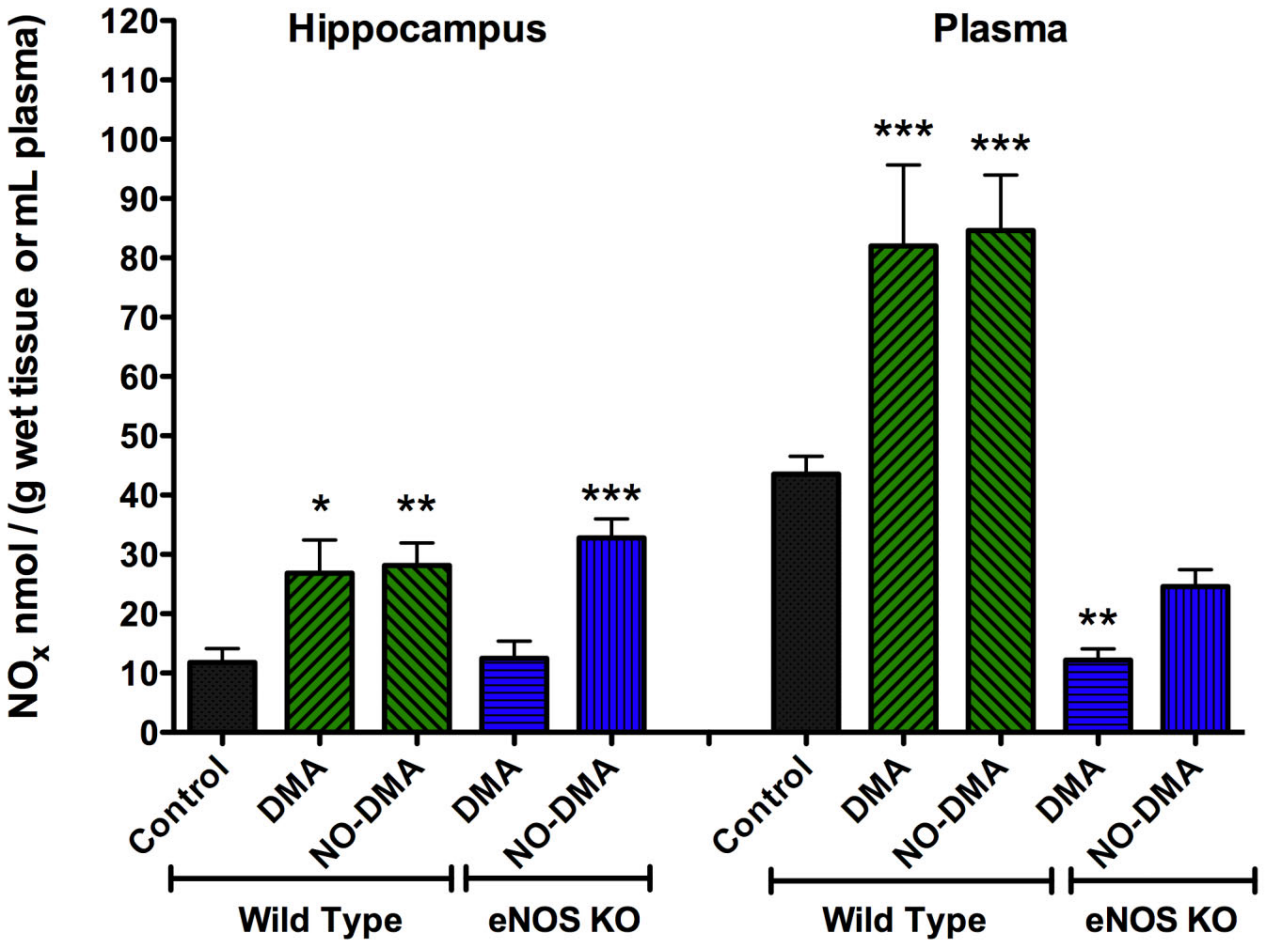
Effects of SERM and NO-SERMon NO levels in plasma and brain of WT and eNOS (−/−) mice. Levels of NO were assessed by measuring breakdown products 1 h after i.p. injection of SERMs (2 mg/kg) using chemiluminescence detection. Both DMA and NO-DMA increased levels of NO in WT mice. The diminished response in eNOS (−/−) was significantly attenuated in DMA relative to NO-DMA treated animals. Data show mean and S.E.M. (n = 4–12); * = p<0.05, ** = p<0.01, *** = p<0.001 compared to wild type vehicle control using one-way ANOVA with Dunnett's post hoc test within each group: hippocampus F_(4,40)_ = 7.79, p<0.0001; plasma F_(4, 23)_ = 21.76, p<0.0001.

The brain and plasma bioavailability of DMA (5 mg/kg; i.p.) was measured in C57Bl/6 mice using LC/MS-MS detection. DMA showed substantial blood brain barrier permeability (Figure S3). Brain and plasma NO levels were assessed by measuring metabolic oxidation products, NO_2_^−^and NO_3_^−^ (NO_x_), using chemiluminescence detection. One hour after injection of DMA or NO-DMA (2 mg/kg), there was a greater than twofold elevation of NO_x_ in the hippocampus and plasma (Fig. 5). To assess the relative contribution of eNOS, either DMA or NO-DMA was administered to eNOS(−/−) animals. eNOS KO mice have lower circulating levels of NO as expected in the absence of the major endogenous cardiovascular source of NO. In these animals, NO-DMA was found to increase NO_x_ significantly, relative to DMA, in both hippocampus and plasma. Collectively, these data support the hypothesis that SERMs activate eNOS, elevating circulating NO_x_ levels. In the presence of eNOS, the contribution of direct release of NO from NO-DMA does not substantially differ from DMA alone, compatible with reports on relatively low fluxes of NO from low doses of mononitrate drugs, while, in eNOS(−/−) mice, without the large background of eNOS-derived NO_x_, the contribution of the NO-donor nitrate functionality of NO-DMA is significant.

### NO-SERM activates both the intrinsic and extrinsic anticoagulant pathways

**Figure 6:**
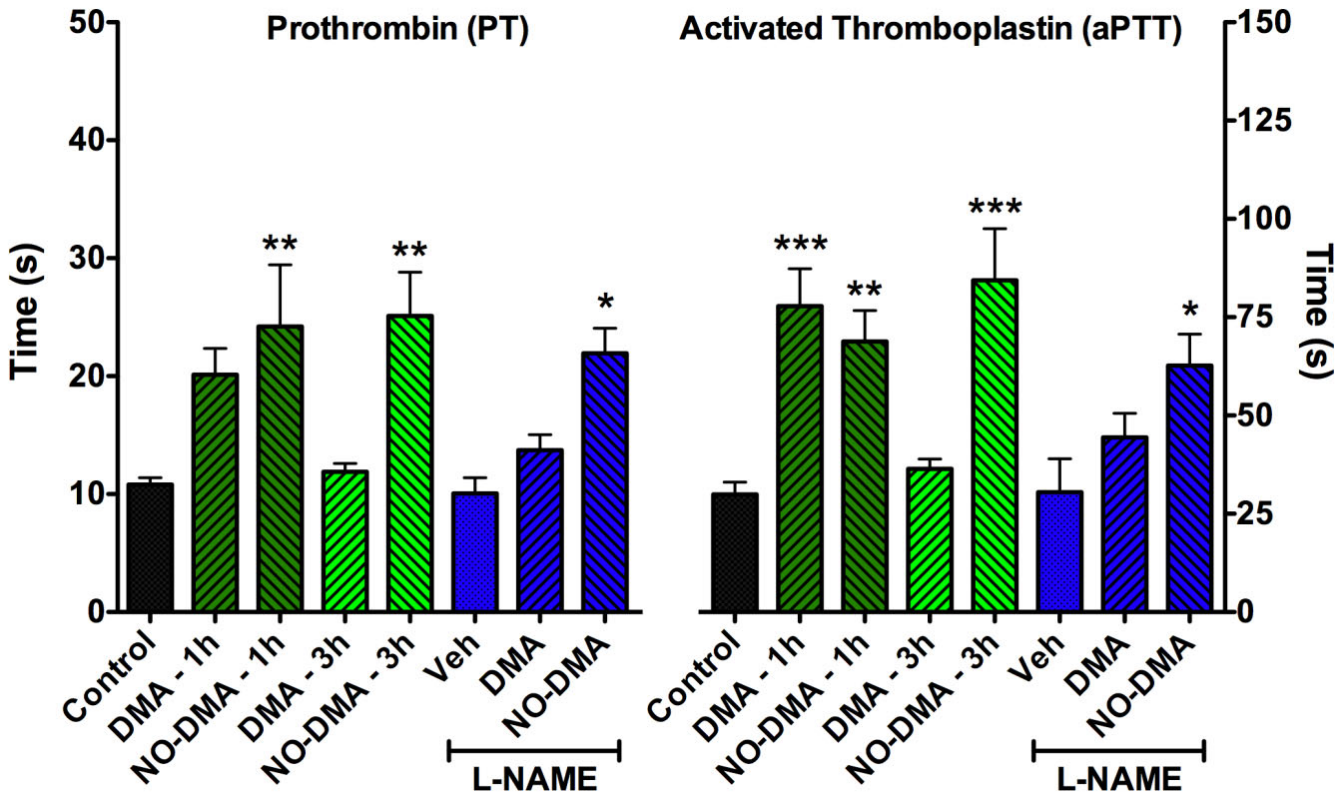
Effect of SERMs and NO-SERM on intrinsic (PTT) and extrinsic (aPTT) clotting cascades. Blood was collected by cardiac puncture and PTT and aPTT were determined 1 or 3 h after i.p. drug administration (2 mg/kg). The anticoagulant activity of DMA was shorter lived than that of NO-DMA and did not persist in the presence of NOS blockade. Data show mean and S.E.M. (n = 2–8); * = p<0.05, ** = p<0.01, *** = p<0.001 compared to wild type vehicle control using one-way ANOVA with Dunnett's post hoc test within each group: PT F_(7,40)_ = 4.33, p = 0.001; aPTT F_(7,37)_ = 6.15, p<0.0001.

Prothrombin (PT) and activated thromboplastin times (aPTT), both pathways associated with the coagulation cascade, were evaluated for DMA and NO-DMA to indicate the potential antithrombotic actions associated with direct release and NOS-mediated release of NO. At 1 h, both DMA and NO-DMA significantly increased aPTT, while only NO-DMA significantly increased PT, though DMA showed a clear trend (Fig. 6). After 3 h, the effect of DMA had decreased to that of control samples, whereas the effect of NO-DMA remained significantly higher than control, compatible with slow release of NO from the NO-donor nitrate functionality. To test the anticoagulant activity of compounds in models of impaired NOS function, mice injected with L-NAME (50 mg/kg, 30 min prior to collection) were administered DMA or NO-DMA (2 mg/kg, 1 h prior to collection), and NO-DMA alone showed significantly increased anticoagulation compared to control in both PT and aPTT.

## Discussion

The benzothiophene SERM, raloxifene, has demonstrated significant beneficial effects on bone and lipid profiles in postmenopausal women [14], [15], [18], in addition to antiestrogenic activity in gynecological tissues [16], [17], and potential positive effects in the CNS [9], [20]. Clinical studies on raloxifene have yielded positive results on cognitive skills in women and elderly men [18], [59], [60], [61], [62], [63], reflecting related observations in both male and female rats [64], [65], [66], [67]. The benzothiophene SERMs, raloxifene and DMA, have many characteristics of an ideal SERM, however, thrombotic side effects need to be alleviated. Relevant to design of an ideal SERM is the interplay between classical ER-mediated transcriptional signaling and emerging mechanisms of rapid extranuclear signaling, such as via GPR30 [49], [68], [69]. Estrogen and SERM induced neuroprotection has been reported to be mediated via rapid signaling, for example, via PI3K/Akt and Src/ERK/CREB [70], [71]. Associated with these kinase pathways, are important roles for NO in signaling via activation of NOS [22], [23]. Therefore, the lack of response to estrogenic therapy with age could well be linked to the known endothelial dysfunction associated with aging and decreased eNOS activity. The SERMs studied herein, DMA, FDMA, and NO-DMA, presented an opportunity to probe mechanism and to provide a preliminary test of the concept that an NO-SERM would circumvent loss of endogenous NOS, and especially eNOS, function.

DMA elicited neuroprotective activity in primary neurons subject to ischemia-reperfusion injury, with the novel observation that neuroprotection was mediated via GPR30 linked to downstream PI3K/Akt, Src, and ERK pathways. FDMA an ERα/ERβ ligand, with similar high affinity was without effect, supporting the lack of dependence on classical ER signaling in this model system. The neuroprotective activity of NO-DMA mirrored that of DMA.

Observations were extended to restoration of cognitive function in response to cholinergic insult, a paradigm which reflects the basis of current symptomatic therapy of AD [72], and provides a test of drugs for early AD and mild cognitive impairment (MCI) [73]. Amnestic MCI is a putative prodromal stage of Alzheimer's disease and chemical amnesia has frequently been induced in human and animal models in drug discovery and development using cholinergic blockade with scopolamine [74]. Benzothiophene SERMs, including DMA, were observed to restore the cognitive deficit induced in mice by scopolamine, although again FDMA was without effect. The dependence of SERM action on eNOS activation was demonstrated by pharmacological NOS inhibition, both in the presence and absence of scopolamine, and the involvement of the endothelial form of NOS was determined conclusively by study in eNOS(−/−) mice. NO-DMA was observed to restore cognition in the absence of eNOS activity, confirming the ability of exogenous NO to circumvent eNOS dysfunction.

The concerted actions of eNOS and nNOS are required for normal LTP in the hippocampus [75]. Therefore the activity of DMA and NO-DMA was studied in hippocampal slices to determine if in addition to cognitive rescue, these SERMS were able to elicit synaptic rescue. The 3×Tg Alzheimer's transgenic mice represent a more complete model of the disease than some other strains, with progressive production of Aβ in relevant brain areas, plaque and tangle pathology comparable to those observed in AD patients, and synaptic transmission and LTP impairment at 6 months of age [56], [76]. We chose to study synaptic function in 3×Tg mice at 16 months, representing a model of aging combined with substantial Aβ and tau neuropathology. Gratifyingly, DMA was observed to restore synaptic deficits, measured by LTP in 3×Tg hippocampal slices; and this activity was again dependent upon GPR30 and retained by NO-DMA.

The actions of estrogens and SERMs are relatively well studied in relaxation of blood vessels and are mediated both by endothelium-independent and -dependent mechanisms, the latter involving activation of eNOS. Both ER isoforms and GPR30 have been implicated in these actions [77], and recently a connection between GPR30 activation and subsequent eNOS-dependent vasodilation has been demonstrated in rat models [78]. The vascular effects of raloxifene have been well established in conduit arteries, and recently in resistance arteries [79], supporting therapeutic benefit in preserving endothelial function. Raloxifene was first reported to induce vasodilation by an ER and eNOS-dependent pathway [80], and subsequently shown to activate eNOS via PI3K/Akt [23]. In rat aorta, vasodilation was observed in response to all benzothiophene SERMs tested. As expected, relaxation induced by DMA was largely, but not completely, endothelium and eNOS-dependent. NO-DMA retained equivalent potency to DMA even in the absence of endothelium and eNOS activity; again demonstrating the ability of exogenous NO to circumvent eNOS dysfunction.

NO has been implicated in directly reducing clotting [25], therefore an NO-SERM might be expected to have enhanced anti-thrombotic activity. The ability of NO-DMA to reduce coagulation by increasing both PT and aPTT suggests this approach can address the risk of thromboembolic events associated with SERM therapeutics. Further, NO-DMA retained significant anticoagulant activity in both pathways, even three hours after administration, compatible with the concept of nitrates as sources of low flux NO release. Again, in contrast to DMA, in the face of NOS inhibition, NO-DMA showed significant activity.

Menopause has been associated with dementia, depression, and anxiety, and the management of such effects with safe and effective hormone therapies is a largely unmet need [81], [82]. The age-adjusted AD mortality rate is higher in women [83]. Several reports support ERT as a means of improving and maintaining levels of cognitive function, and potentially reducing the risk of AD, in accord with proposed roles for estrogen in the brain in protection, growth and differentiation of neurons [4], [84], [85], [86]. However, serious adverse events such as increased risk of breast cancer and stroke associated with estrogen itself, commend the use of an ideal SERM as an alternative approach. Such ideal SERM design requires better understanding of the role of estrogen receptor signaling in two key areas where estrogenic activity is beneficial, the brain and cardiovascular system, and strategies to overcome the remaining risk of thromboembolic events.

The known endothelial dysfunction associated with aging, the decreased expression of eNOS in postmenopausal women, and the potential contribution of eNOS to the critical period hypothesis, commend the further study of therapeutic approaches that reinforce NO/cGMP signaling in combination with ER modulation. Other contributions to age-related pathophysiology, in addition to attenuated eNOS expression, have been proposed, including: inhibition of eNOS by elevation of ADMA [87], [88]; depletion of cofactors, notably BH4 [89], [90], [91]; and eNOS polymorphisms [92]. Attenuation of NO-dependent cGMP production by sGC has also been linked directly with Aβ processing [93]. Testing of the NO-SERM concept is informative in revealing the effects of circumventing eNOS dysfunction, however, other approaches can be considered to achieve similar therapeutic actions, such as modulating estrogenic and NO/cGMP signaling separately with individual therapeutic agents [24], [94].

## Conclusions

In summary, a prototype NO-SERM was designed, retaining the ER binding affinity and ER antagonist activity of the parent SERM. The NO-SERM also retained cardiovascular and neuroprotective functions and the ability both to restore cognition after cholinergic challenge and restore synaptic function in an aged AD mouse model. In contrast to the parent SERM, the NO-SERM was able to circumvent NOS inhibition of anticoagulant activity and loss of eNOS function in cardiovascular activity and in restoration of cognition. The comparison of 3 structurally similar SERMs as chemical probes demonstrates a key role for signaling via GPR30 in mediating the beneficial effects of SERMs on synaptic function and neuroprotection. In parallel, the importance of eNOS was confirmed in vasodilation and demonstrated in cognition. In AD in particular, a role is emerging for NO in treating cholinergic deficits [51] and attenuating amyloidogenesis [95], [96]. These findings, coupled with the otherwise excellent safety record of benzothiophene SERMs in the clinic, support further development of NO-SERMs or combination therapies in the peri- and post-menopausal cohort, and hint at utility for this approach in the general population.

## Materials and Methods

### Ethics Statement and Animal Use

Use of animals was approved by the Institutional Animal Care and Use Committee at the University of Illinois at Chicago. All experiments conformed to the Animal Welfare Act, Guide to Use and Care of Laboratory Animals, and the US Government Principles of the Utilization and Care of Vertebrate Animals Used in Testing, Research and Training guidelines on the ethical use of animals.

### Synthesis of NO-DMA and DMA analogues

Synthesis of F-DMA and DMA has been described [42]. Synthesis of NO-DMA (3-(1-(2-(4-(6-hydroxy-2-(4-ydroxyphenyl)benzo[b]thiophen-3-yloxy)phenoxy)ethyl)piperidin-4-yl)propyl nitrate) and full characterization data are supplied in Supporting Information (File S1).

### Primary Neuron Preparation

Primary cultures of dissociated cortical neurons were prepared as follows; briefly, cortices were dissected from E16-18 Sprague-Dawley rats (Charles River). After removal of meninges, cortices were manually dissociated and plated at a density of 10^6^ cells/mL into 96-well plates. Twenty-four hours after plating, cultures were grown in Neurobasal A media supplemented with B27, glutamine and pen/strep, with media changes every 3–4 days. In accordance with published protocols, measured levels of glia in culture after DIV2 are <0.5%. Experiments were performed at DIV 11–12.

### Oxygen Glucose Deprivation (OGD)

The cell media was changed to phenol red-free growing media at least 3 h before OGD. Receptor blockers were added 45 min prior to the start of OGD, and SERMs were added at the start of OGD, with concentrations maintained through media changes. For OGD, the cells were transferred to a hypoxia chamber controlled at 5% CO_2_ and <1% O_2_, and growth media was replaced with a physiological salt solution (in mM: NaCl 116, CaCl_2_ 1.8, MgSO_4_ 0.8, KCl 5.4, NaHCO_3_ 14.7, NaHPO_4_ 1, HEPES 10, pH = 7.4). After 2 h OGD, cells were removed from the chamber and growth media was replaced. After 24 h, cell viability was measured by MTT assay using a Dynex MRX ll micro-plate spectrophotometer.

### Electrophysiology

All experiments used 16-month old male 3×Tg transgenic or age-matched WT background controls. For electrophysiology, mice were rapidly decapitated, and brains were removed into an ice-cold aCSF solution (in mM: NaCl 124, KCl 3.0, KH_2_PO_4_ 1.25, NaHCO_3_ 25.7, D-glucose 10, L-ascorbate 2.0, MgSO_4_ 2.5, and CaCl_2_ 3.3) and sectioned on a tissue chopper into 400 µm sections. Slices were transported to a 37°C solution of aCSF, continuously bubbled with 95% O_2_/5% CO_2_, and allowed to recover at least 60 min before experimentation. After placement of a stimulation electrode in the Schaffer commissural fibers and recording electrodes with 2 M NaCl solution into the stratum radiatum of the CA1 area, stimulus intensity was set to evoke submaximal fEPSP and continuously monitored at 20 s intervals for at least 15 min to establish a stable baseline. SERMs (100 nM) and G15 (100 nM) were prepared fresh in perfusate and started at least 30 min before LTP induction to reach a new baseline after enhancement of basal synaptic transmission was observed [97]. LTP was induced using a theta burst induction protocol by applying 10 bursts of four pulses at 100 Hz with an interburst interval of 200 msec. Resulting fEPSP was monitored at 20 s intervals for ∼60 min post TBS.

### Bioavailability Measurement

All experiments were performed on 6–8 month male C57B1/6 mice (Charles River Laboratory) or eNOS(−/−) animals. Animals were injected (i.p.) at various time points before sacrifice at the doses specified. After euthanasia via CO_2_, plasma was collected, and cortices and hippocampi were collected after PBS perfusion and stored at −80°C. Tissue samples were weighed and extracted with methanol. Plasma samples were extracted with cold acetonitrile. Quantitative analysis of drug concentrations in plasma and brain used internal standards spiked into plasma and brain homogenates before liquid extraction with acetonitrile or methanol, and separation and measurement was performed with LC-MS/MS tandem mass spectrometry. The supernatant (deproteinized) was analyzed by chemiluminescence with SIEVERS 280i nitric oxide analyzer.

### Behavior

All experiments were performed either on 6–8 month old male C57B1/6 mice or eNOS(−/−) animals. Scopolamine (1 mg/kg) or L-NAME (50 mg/kg) were injected (i.p.) 30 min prior to training, while SERMs (2 mg/kg) were injected 20 min prior to training. Mice were placed in the light compartment of the light/dark box, and as soon as they entered the dark compartment, they received an electric shock (0.5 mA, 60 Hz for 2 seconds). This training was repeated until latency to enter the dark side reached 300 s. At 24 h post-training, animals were individually placed in the light compartment and the latency to enter the dark compartment was recorded with a 300 s cutoff. Details of this widely used assay of behavior have been discussed previously for NO-donor compounds [53], [54], [55].

### Aortic Ring Relaxation

Isolated rings of aortae from male Sprague-Dawley rats (250–300 g) were prepared for isometric tension measurements and were equilibrated for 1 h at an optimal resting tension of 10 mN. Tissues were contracted submaximally with 0.3 µM phenylepinephrine, and after 10–15 m cumulative concentration-response curves for SERMs (0.01–30 µM) were obtained. Some aortic ring preparations were denuded of the endothelium, or were treated with 100 µM L-NAME, prior to assessment of SERM-induced relaxation responses.

### Anticoagulation

SERMs were injected (i.p.) into 6–8 month old male C57Bl/6 mice 1 h before blood collection, and L-NAME was injected (i.p.) 30 min before blood collection. The mouse was sedated by exposing it to CO_2_ until unconscious and blood was collected in 4.5 mL BD Vacutainer Glass Evacuated Blood Collection Tubes (with 0.105 M buffered sodium citrate) by cardiac puncture. The blood samples were centrifuged at 300 g for 30 min to separate the plasma from the blood, and then 200 uL of plasma was taken from each sample and the PT and aPTT were measured using an ACL 7000 Coagulation Analyzer. The analyzer injects 125 µM of PT-fibrinogen (lyophilized rabbit brain calcium thromboplastin with stabilizers, polybrene, buffer and preservatives) to 75 µL of plasma sample to measure the PT, and 75 µL of synthAFAX (0.025 M)+100 µL of CaCl_2_ (0.02 M) were added to 75 µL of plasma to measure aPTT. The analysis was at 37°C using a photo sensor at λ = 671 nm.

## Supporting Information

File S1 **Structures & Synthesis.**
(DOCX)

Figure S1 **G15 has no effect on LTP in C57Bl/6 mice.**
(DOCX)

Figure S2 **sGC inhibition blocks the effect of DMA on LTP in 3×Tg mice.**
(DOCX)

Figure S3 **SERM bioavailability in plasma and CNS of WT and eNOS (−/−) mice.**
(DOCX)
