# Supplementary material for: An NO Donor Approach to Neuroprotective and Procognitive Estrogen Therapy Overcomes Loss of NO Synthase Function and Potentially Thrombotic Risk
Source: PLoS One. 2013 Aug 16;8(8):e70740. doi: 10.1371/journal.pone.0070740 (PMC3745399; doi:10.1371/journal.pone.0070740)
Supplement: File S1 — Structures & Synthesis. (DOCX) [file pone.0070740.s001.docx]

**Structures**

**
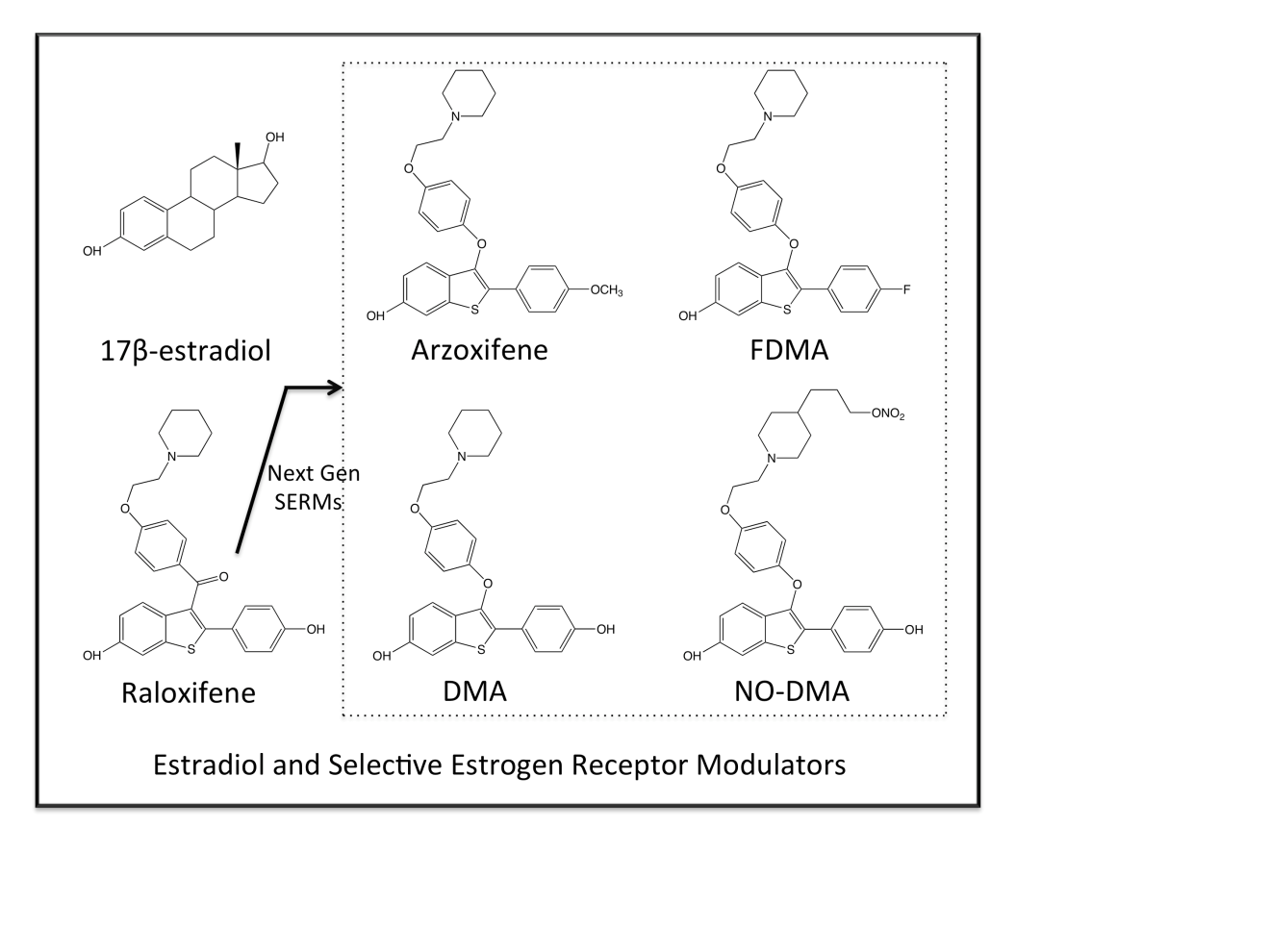
**

**Synthesis**

**Synthesis of NO-DMA compound 8**

^1^H and ^13^C NMR spectra were obtained with Bruker Ultrashield 400 MHz and Advance 300 MHz spectrometers. Chemical shifts are reported as δ values in parts per million (ppm) relative to tetramethylsilane (TMS) for all recorded NMR spectra. All reagents and solvents were obtained commercially from Acros, Aldrich, and Fluka and were used without purification.

Starting material **1** was synthesized from commercial available 6-methoxy-2-(4-methoxy phenyl)benzo[b]thiophene according to a published method in 6 steps.^1^ Demethylation of **4** gave complex mixture, might be due to the incompatibility of Lewis acid with organic nitrate. NO-DMA **8** was obtained by the reaction of **5** with silver nitrate in low yield (method A); or by using tetrabutyl ammonium nitrate as nitration reagent, the yield was improved dramatically, but the method suffered from repetitive column purifications to remove ammonium salt (method B); alternatively, the phenol groups of **5** were acetylated and the bromide **6** was converted nitrate **7** using silver salt, **8** was finally obtained with good purity by de-acetylation of **7** in high yield (method C).

**Compound 2.** Compound **1** (2.6g, 5.3mmol), 4-piperidinepropanol (1.0g, 6.9mmol) were dissolved in anhydrous acetonitrile (100mL) and anhydrous K_2_CO_3_ (2.9g, 21.5mmol) was added. The reaction mixture was refluxed for 6hrs and cooled to room temperature. After filtration, solvent was removed, the residue was dissolved in DCM (150mL) and washed with water (2×50mL). Organic phase separated and concentrated. The residue was purified by column chromatography (DCM/MeOH 20:1, 2.9g, quantitative yield). ^1^H-NMR (Acetone-d_6_, 400 MHz): δ 7.66-7.69(m, 2H), 7.42(d, 1H, *J*=2.0Hz), 7.24(d, 1H, *J*=8.8Hz), 6.81-6.93(m, 7H), 3.97(t, 2H, *J*=6.0Hz), 3.84(s, 3H), 3.76(s, 3H), 3.51(t, 2H, *J*=6.4Hz), 2.88-2.91(m, 2H), 2.63(t, 2H, *J*=6.0Hz), 1.95-2.01(m, 2H), 1.60-1.62(m, 2H), 1.46-1.53(m, 2H), 1.21-1.27(m, 5H); ^13^C-NMR (Acetone-d_6_, 100 MHz): 160.23, 159.01, 155.19, 152.34, 140.57, 137.56, 129.32, 128.77, 127.06, 125.78, 122.64, 116.95, 116.26, 115.28, 115.05, 106.36, 67.30, 62.64, 58.28, 55.90, 55.52, 55.13, 36.32, 33.62, 33.31, 30.90.

**Compound 3**. Compound **2** (1.1g, 2.0 mmol) was dissolved in chloroform (25mL), CBr_4_ (2.7g, 8.0mmol) and polymer bound Ph_3_P (2.6g, about 3mmol/g) were added, the reaction mixture was stirred at r.t. overnight. After filtration, solvent was removed, and the residue was purified by column chromatography (DCM/AcOEt/MeOH 3:1:0.2, 1.1g, 89%). ^1^H-NMR (Acetone-d_6_, 400 MHz): 7.68-7.72(m, 2H), 7.46(d, 1H, *J*=2.0Hz), 7.25(d, 1H, *J*=8.8Hz), 6.84-6.97(m, 7H), 4.00(t, 2H, *J*=6.0Hz), 3.87(s, 3H), 3.80(s, 3H), 3.47(s, 2H, *J*=6.8Hz), 2.91-2.94(m, 2H), 2.67(t, 2H, *J*=6.0Hz), 1.99-2.04(m, 2H), 1.82-1.89(m, 2H), 1.62-1.65(m, 2H), 1.32-1.37(m, 2H), 1.15-1.24(m, 3H); δ ^13^C-NMR (Acetone-d_6_, 100 MHz): 160.35, 159.12, 155.29, 152.43, 140.64, 137.63, 129.38, 128.82, 127.10, 125.63, 122.68, 117.00, 116.36, 115.37, 115.12, 106.43, 67.43, 58.30, 55.96, 55.58, 55.06, 35.83, 35.78, 34.98, 33.22, 30.98.

**Compound 4.** Compound **3** (110mg, 0.18mmol) was dissolved in anhydrous acetonitrile (3mL), AgNO_3_ (306mg, 1.8mmol) was added. The reaction mixture was stirred at r.t. for 24h. Solvent was removed and the residue was purified by column chromatography (DCM/AcOEt/MeOH 2:1:0.25, 85mg, 79%). ^1^H-NMR (Acetone-d_6_, 400 MHz): δ7.66-7.72(m, 2H), 7.43(d, 1H, *J*=2.0Hz), 7.25(d, 1H, *J*=8.8Hz), 6.82-6.95(m, 7H), 4.49(t, 2H, *J*=6.8Hz), 3.98(t, 2H, *J*=6.0Hz), 3.85(s, 3H), 3.77(s, 3H), 2.89-2.92(m, 2H), 2.64(t, 2H, *J*=2.0Hz), 1.97-2.02(m, 2H), 1.67-1.73(m, 2H), 1.61-1.64(m, 2H), 1.13-1.34(m, 5H); ^13^C-NMR (Acetone-d_6_, 100 MHz): δ160.30, 159.07, 155.25, 152.39, 140.62, 137.61, 129.36, 128.81, 127.09, 125.62, 122.67, 116.99, 116.32, 115.32, 115.09, 106.41, 74.69, 67.40, 58.27, 55.94, 55.56, 54.99, 36.03, 33.09, 24.64.

**Compound 5**. Compound **3** (240mg, 0.39mmol) was dissolved in DCM (5mL), HCl ether solution (2.0M, 1.0mL) was added. The reaction mixture was stirred at 4°C for 2hr. Solvent was removed, the residue was re-dissolved in DCM (10mL), cooled in an ice-water bath, BBr_3_ in DCM (1.0M, 1mL) was added dropwise. The reaction mixture was stirred at 4°C for 3hr and then at r.t. for another hour. The reaction mixture was poured into ice water (50mL), neutralized with NaHCO_3_ and extracted with AcOEt (3×30mL). Organic solution was combined and concentrated, the residue was purified by column chromatography (DCM/MeOH 50:3, 160mg, 70%). ^1^H-NMR (Acetone-d_6_, 400 MHz): δ 7.59-7.62(m, 2H), 7.32(d, 1H, *J*=2.0Hz), 7.19(d, 1H, *J*=8.8Hz), 6.83-6.89(m, 7H), 4.06(t, 2H, *J*=5.9Hz), 3.47(t, 2H, *J*=6.7Hz), 3.01-3.04(m, 2H), 2.77(t, 2H, *J*=5.9Hz), 2.11-2.17(m, 2H), 1.82-1.90(m, 2H), 1.65-1.67(m ,2H), 1.22-1.38(m, 5H); ^13^C-NMR (Acetone-d_6_, 100 MHz): δ158.14, 156.63, 154.99, 152.52, 140.31, 137.46, 129.41, 128.06, 126.60, 124.59, 122.67, 116.97, 116.54, 116.30, 115.64, 108.79, 66.85, 58.04, 54.83, 35.61, 35.52, 34.95, 32.68, 30.90.

**Compound 6**. Compound **5** (300mg, 0.52mmol) was dissolved in DCM(10mL), pyridine (0.84mL, 10.3mmol) and Ac_2_O (0.34mL, 3.6mmol) were added, the reaction mixture was stirred at r.t. overnight. Solvent was removed, the residue was re-dissolved in ethyl acetate(50mL) and washed with water, organic solution was separated and concentrated, the residue was purified by column chromatography, product was obtained as slightly yellow foam (DCM/MeOH 30:1, 298mg, 87%). ^1^H-NMR (Acetone-d_6_, 400 MHz): δ 7.83 (d, 2H, *J*=8.4Hz), 7.74(d, 1H, *J*=2.0Hz), 7.40(d, 1H, *J*=8.8Hz), 7.19(d, 2H, *J*=8.8Hz), 7.10(dd, 1H, *J*=8.8Hz, 2.0Hz), 6.86-6.93(m, 4H), 4.05(t, 2H, *J*=6.0Hz), 3.47(t, 2H, *J*=6.8Hz), 2.95-2.98(m, 2H), 2.71(t, 2H, *J*=6.0Hz), 2.29(s,3H), 2.26(s, 3H), 2.06-2.11(m, 2H), 1.82-1.89(m, 2H), 1.64-1.66(m, 2H), 1.32-1.38(m, 2H), 1.20-1.28(m, 3H); ^13^C-NMR (Acetone-d_6_, 100 MHz): 169.75, 169.46, 155.42, 152.20, 151.79, 149.90, 141.64, 136.84, 132.49, 130.24, 129.33, 129.24, 123.25, 122.81, 120.61, 117.13, 116.89, 116.46, 67.17, 58.12, 54.93, 35.69, 35.66, 34.98, 32.96, 30.94, 20.93.

**Compound 7**. Compound **6** (280mg, 0.45mmol) was dissolved in anhydrous acetonitrile (15mL), AgNO_3_ (1.1g, 1.8mmol) was added. The reaction mixture was stirred at r.t. for 24h. After filtration, solvent was removed and the residue was purified by column chromatography, product was obtained as slightly yellow foam (DCM/MeOH 30:1, 190mg, 70%). ^1^H-NMR (Acetone-d_6_, 400 MHz): δ^1^H-NMR (Acetone-d_6_, 400 MHz): δ 7.81(d, 2H, *J*=8.8Hz), 7.71(d, 1H, *J*=2.0Hz), 7.38(d, 1H, *J*=8.8Hz), 7.17(d, 2H, *J*=8.8Hz), 7.08(dd, 1H, *J*=8.8Hz, 2.0Hz), 6.82-6.92(m, 4H), 4.48(t, 2H, *J*=6.8Hz), 4.00(t, 2H, *J*=6.0Hz), 2.92-2.95(m, 2H), 2.68(t, 2H, *J*=6.0Hz), 2.27(s, 3H), 2.24(s,3H), 2.02-2.04( m, 2H), 1.61-1.74(m, 4H), 1.16-1.31(m, 5H); ^13^C-NMR (Acetone-d_6_, 100 MHz): δ 169.69, 169.41, 155.34, 152.12, 151.70, 149.82, 141.58, 136.77, 132.44, 130.19, 129.25, 129.19, 123.18, 122.77, 120.53, 117.08, 116.82, 116.38, 74.65, 67.09. 58.07, 54.85, 35.83, 32.96, 32.79, 24.58, 20.93.

**Compound 8**. Method A: Compound **5** (55mg, 0.095mmol) was dissolved in anhydrous acetonitrile (2mL), AgNO_3_ (160mg, 0.95mmol) was added. The reaction mixture was stirred at r.t. overnight. After filtration, solvent was removed and the residue was purified by column chromatography (DCM/AcOEt/MeOH 3:1:0.3), product was obtained as white foam (15mg, 28%). Method B: Compound **5** (100mg, 0.17mmol) was dissolved in anhydrous acetonitrile (5mL), tetrabutylammonium nitrate (523mg, 1.7mmol) was added. The reaction mixture was heated at 70°C for 8hrs. Solvent was removed and the residue was purified repeatedly by column chromatography (DCM/AcOEt/MeOH 3:1:0.3) three times, product was obtained as white foam (68mg, 70%). Method C: Compound **7** (130mg, 0.2mmol) was dissolved in a mixture of THF-MeOH-H_2_O (4.5mL, 2:2:0.5), K_2_CO_3_ (79mg, 0.58mmol) was added. The reaction mixture was stirred at r.t. for 1.5hr. After filtration and concentration, the residue was purified by column chromatography (DCM/MeOH 20:1), product was obtained as white foam (103mg, 91%).

^1^H-NMR (Acetone-d_6_, 400 MHz): δ 7.60(d, 2H, *J*=8.8Hz), 7.31(d, 1H, *J*=2.0Hz), 7.19(d, 1H, *J*=8.8Hz), 6.84-6.90(m, 7H), 4.53(t, 2H, *J*=6.7Hz), 4.03(t, 2H, *J*=5.9Hz), 2.95-2.98(m, 2H), 2.70(t, 2H, *J*=5.9Hz), 1.98-2.04(m, 2H), 1.73-1.77(m, 2H), 1.66-1.69(m, 2H), 1.19-1.35(m, 5H); ^13^C-NMR (Acetone-d_6_, 100 MHz): δ158.14, 156.62, 155.11, 152.50, 140.36, 137.49, 129.43, 128.12, 126.63, 124.64, 122.71, 116.98, 116.56, 116.29, 115.47, 108.81, 74.69, 67.12, 58.21, 54.93, 35.94, 33.03, 32.86, 24.63.

**Reference**

1. Liu, H.; Qin, Z.; Thatcher, G. R.; Bolton, J. L. Uterine peroxidase-catalyzed formation of diquinone methides from the selective estrogen receptor modulators raloxifene and desmethylated arzoxifene. *Chem Res Toxicol* **2007,** 20, 1676-84.
