# Supplementary material for: An NO Donor Approach to Neuroprotective and Procognitive Estrogen Therapy Overcomes Loss of NO Synthase Function and Potentially Thrombotic Risk
Source: PLoS One. 2013 Aug 16;8(8):e70740. doi: 10.1371/journal.pone.0070740 (PMC3745399; doi:10.1371/journal.pone.0070740)
Supplement: Figure S1 — G15 has no effect on LTP in C57Bl/6 mice. (DOCX) [file pone.0070740.s002.docx]

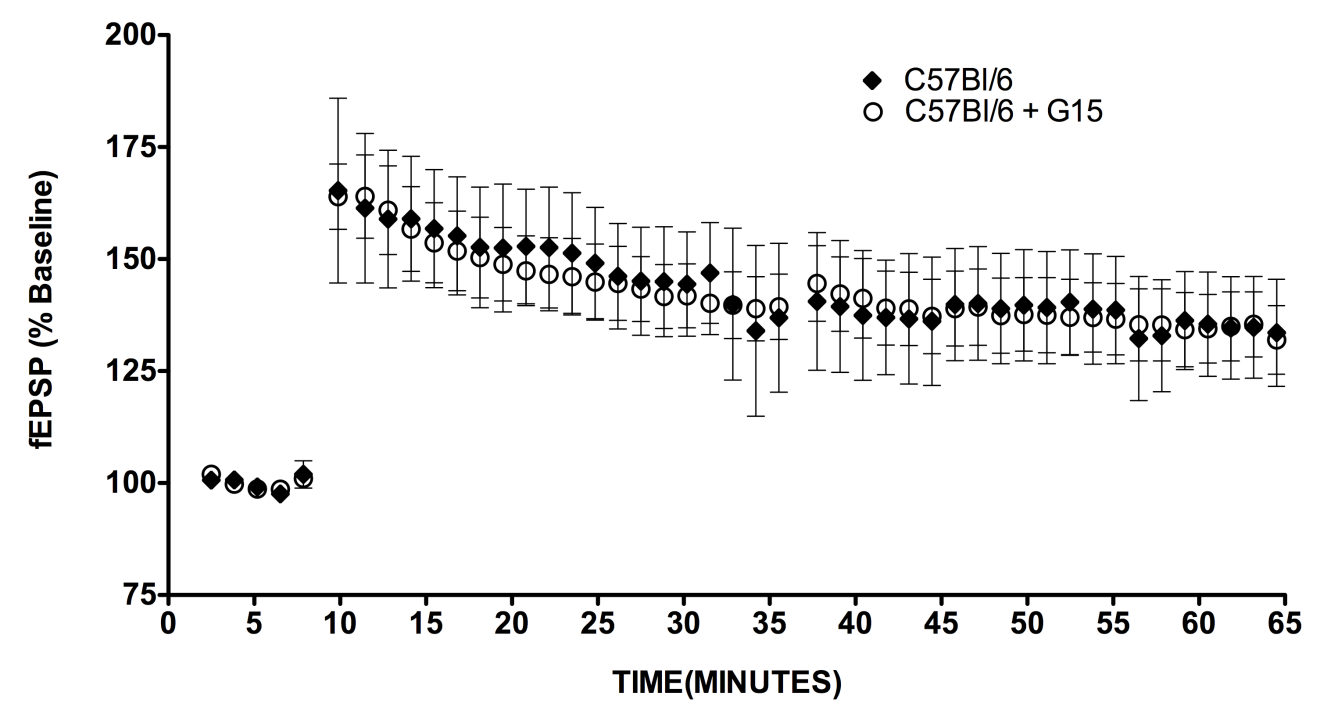


**Figure S1: G15 has no effect on LTP in C57Bl/6 mice.** LTP was measured after TBS in the CA1 region of hippocampal sections from 8 mo male C57Bl/6 mice. G15 (100 nM) was added 30 min prior to TBS and continued throughout. G15 had no effect on LTP, which reached levels equal to WT background in Figure 3 for both controls and G15. Data show mean and s.e.m. normalized to baseline (n=5-6).
