# Supplementary material for: An NO Donor Approach to Neuroprotective and Procognitive Estrogen Therapy Overcomes Loss of NO Synthase Function and Potentially Thrombotic Risk
Source: PLoS One. 2013 Aug 16;8(8):e70740. doi: 10.1371/journal.pone.0070740 (PMC3745399; doi:10.1371/journal.pone.0070740)
Supplement: Figure S2 — sGC inhibition blocks the effect of DMA on LTP in 3×Tg mice. (DOCX) [file pone.0070740.s003.docx]

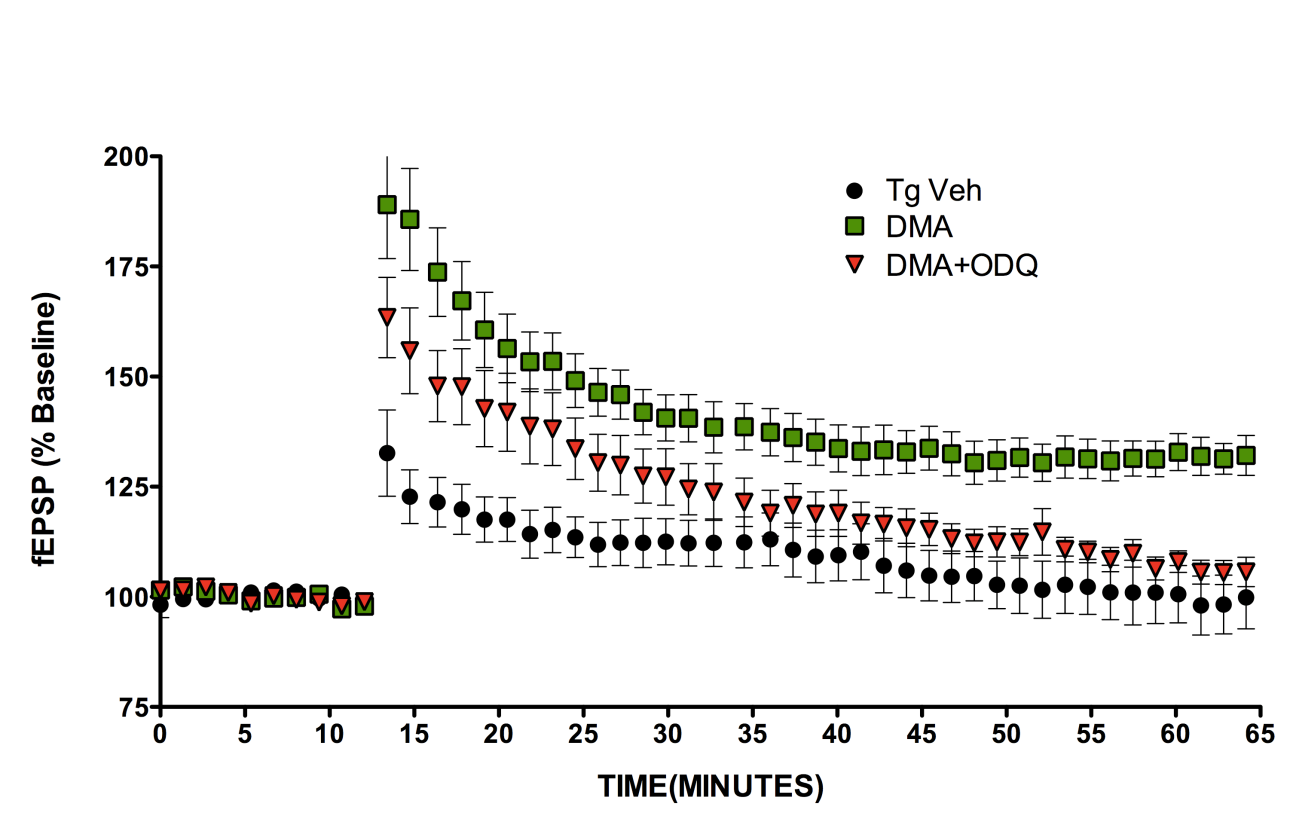


**Figure S2: ODQ blocks effect of DMA on LTP in 3xTg mice.** LTP was measured in the CA1 region of hippocampal sections from 16 mo male 3xTg mice using the method detailed in manuscript, with the exception of a variable interburst interval during TBS. These data are presented to provide support in showing dependence on NO/cGMP for SERM action on LTP, but the variability in TBS interval prevents direct comparison to Figure 3 in the main manuscript. ODQ (10 nM) was added 30 min prior to TBS and continued throughout. Data show mean and s.e.m. normalized to baseline (n=9-10).


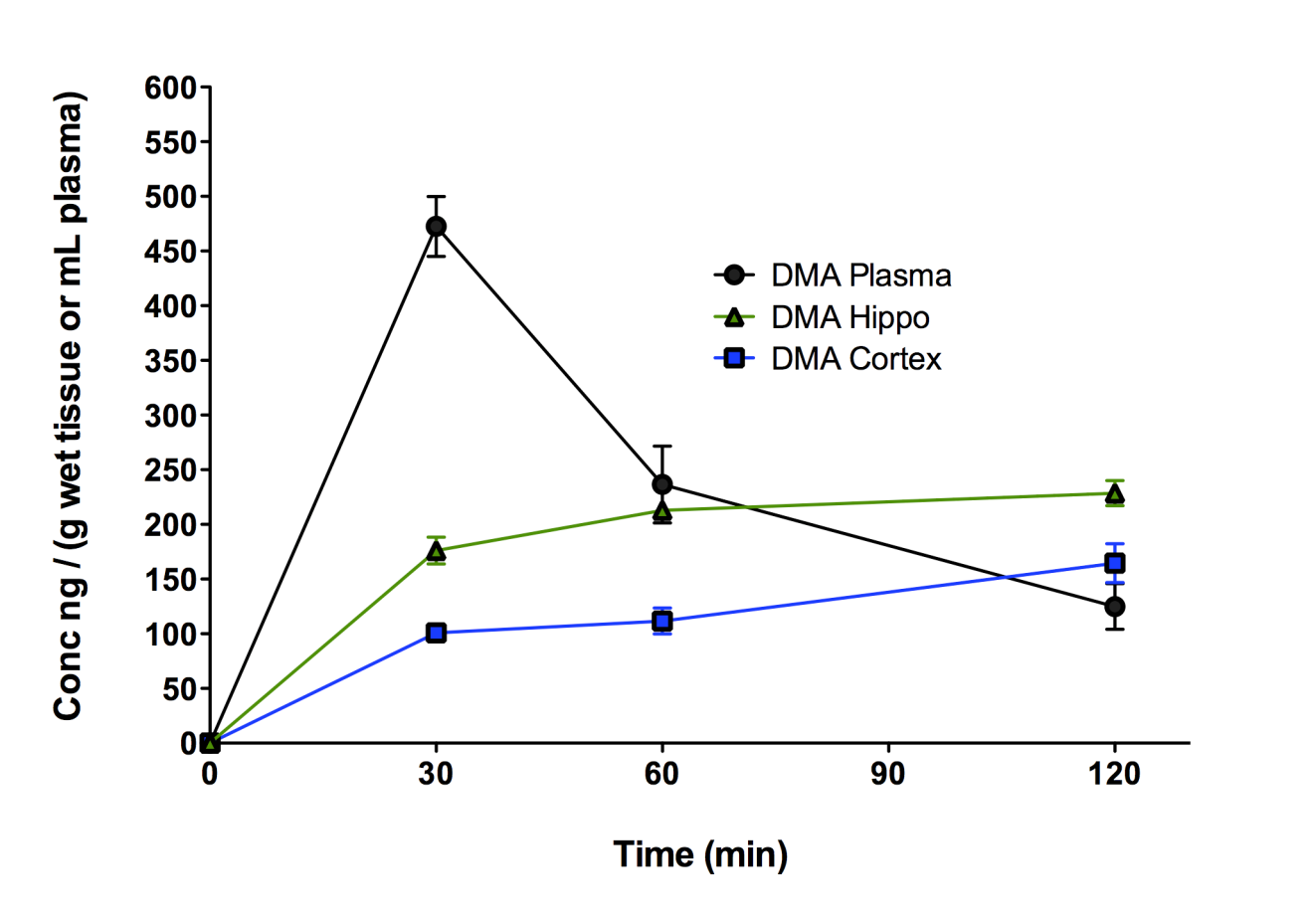


**Figure S3: SERM bioavailability in plasma and CNS of WT and eNOS (-/-) mice.** Bioavailability was assessed using LC/MS-MS after liquid extraction with internal standard after i.p. injection of 5 mg/kg DMA. DMA shows substantial bioavailability with preferential retention in the hippocampus up to 2 h after administration. Data show mean and s.e.m. (n=4).
