# Supplementary material for: An NO Donor Approach to Neuroprotective and Procognitive Estrogen Therapy Overcomes Loss of NO Synthase Function and Potentially Thrombotic Risk
Source: PLoS One. 2013 Aug 16;8(8):e70740. doi: 10.1371/journal.pone.0070740 (PMC3745399; doi:10.1371/journal.pone.0070740)
Supplement: Figure S3 — SERM bioavailability in plasma and CNS of WT and eNOS (−/−) mice. (DOCX) [file pone.0070740.s004.docx]

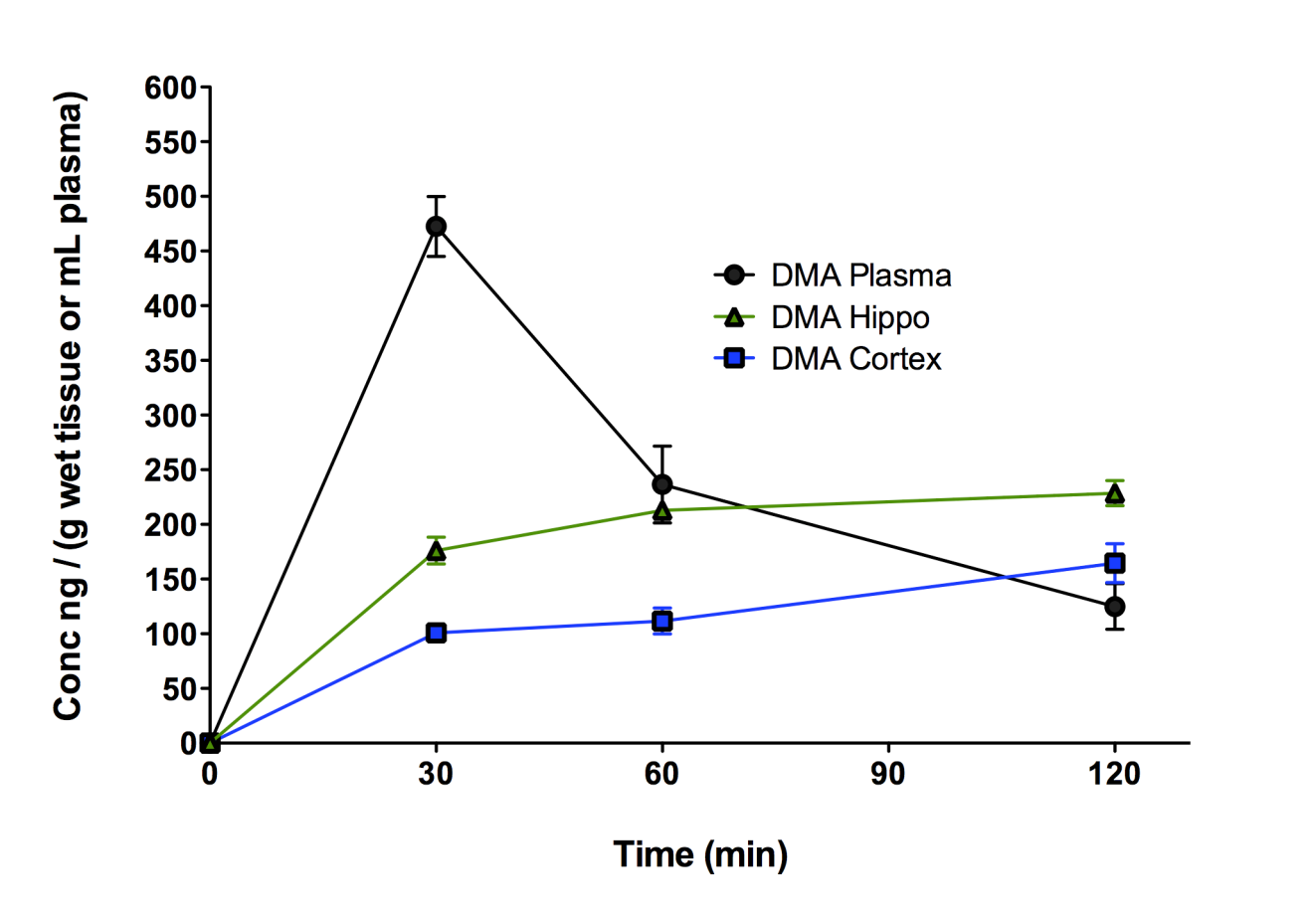


**Figure S3: SERM bioavailability in plasma and CNS of WT and eNOS (-/-) mice.** Bioavailability was assessed using LC/MS-MS after liquid extraction with internal standard after i.p. injection of 5 mg/kg DMA. DMA shows substantial bioavailability with preferential retention in the hippocampus up to 2 h after administration. Data show mean and s.e.m. (n=4).
